# Exposure to the Environmental Toxins Microcystin-LR and Roundup^®^ Causes Cellular Damage in the Hepatopancreas of the White River Crayfish (*Procambarus acutus*)

**DOI:** 10.3390/toxics14080712

**Published:** 2026-08-12

**Authors:** Mona Khalil, Antonillamarein E. Hanna, Brandon T. Kenaya, Amira S. Abuhmoud, Sarah A. Magid, Miranda J. Jasinski, Shaza Ahmed, Andrew A. Bosah, Sabrina A. Cacanindin, Oswaldo Celestino Escobar, Yara A. El-Sheikh, Evelyn M. Rihacek, Kendra R. Evans, Gregory M. Grabowski, Levi Storks, Rachelle M. Belanger

**Affiliations:** 1 Biology Department, University of Detroit Mercy, Detroit, MI 48221, USA; mkhalil1@udmercy.edu (M.K.); hannaan@udmercy.edu (A.E.H.); kenayabt@udmercy.edu (B.T.K.); abuhmoam@udmercy.edu (A.S.A.); magidsa@udmercy.edu (S.A.M.); jasinsmj@udmercy.edu (M.J.J.); grabowgm@udmercy.edu (G.M.G.); storksle@udmercy.edu (L.S.); 2 Department of Chemistry and Biochemistry, University of Detroit Mercy, Detroit, MI 48221, USA; shaza0521@gmail.com (S.A.); andrewbosah@gmail.com (A.A.B.); sabrina_181@outlook.com (S.A.C.); celestino.osw@gmail.com (O.C.E.); 2yara.elsheikh@gmail.com (Y.A.E.-S.); emrihacek@gmail.com (E.M.R.); evanskr@udmercy.edu (K.R.E.)

**Keywords:** herbicide, cell death, apoptosis, cyanotoxin, morphology, toxicity

## Abstract

Aquatic ecosystems are routinely exposed to natural and anthropogenic stressors, and when multiple pollutants co-occur, their synergistic effects may exceed those of individual contaminants. This study evaluated the individual and combined effects of the widely used agrochemical Roundup^®^, whose active ingredient is glyphosate (GLY) and the cyanobacterial toxin microcystin-LR (MC-LR) on the hepatopancreas of crayfish. Following a seven-day exposure to MC-LR (15 ppb), GLY (75 ppb), or their combination (MC-LR + GLY), cellular and morphological alterations were assessed using flow cytometry and histological analyses. MC-LR exposure increased hepatopancreatic vacuole number 3.7-fold and vacuole proportion 6.8-fold relative to controls, whereas GLY increased vacuole proportion 5.2-fold. Combined exposure increased tubule lumen proportion by approximately 65%, while epithelial height remained unchanged. GLY reduced viable (fluorescein diacetate-positive) hepatopancreatic cells by approximately 86%, and both GLY and MC-LR + GLY increased non-viable (propidium iodide-positive) cells by 4.8-fold and 4.3-fold, respectively. MC-LR alone did not significantly affect cell viability. These findings indicate that MC-LR primarily induces structural alterations, whereas GLY causes pronounced cellular cytotoxicity. Although co-exposure did not consistently produce synergistic toxicity, it enhanced tissue remodeling, emphasizing the importance of evaluating environmentally relevant contaminant mixtures and their implications for freshwater ecosystem health.

## 1. Introduction

Environmental toxicology has traditionally examined biological responses to anthropogenic pollutants and natural toxins separately; however, organisms in natural ecosystems are frequently exposed to both simultaneously. Agrochemicals are introduced to aquatic environments through agricultural runoff, drift, atmospheric deposition, leaching from soils, and direct application [1]. These agrochemicals can co-occur with naturally produced toxins such as cyanobacterial metabolites, creating complex exposure scenarios with potentially antagonistic, additive, or synergistic effects [2,3]. Antagonistic interactions occur when the combined effect of two chemicals is less than expected because one chemical reduces or inhibits the activity of the other. This can occur when one substance neutralizes, degrades, or inhibits the action of another, such as by blocking its receptor or interfering with its biological activity [3,4]. Additive interactions occur when the combined effect of two chemicals is equal to the sum of their individual effects [3,4]. In contrast, synergistic interactions occur when the combined effect exceeds the expected additive effect, resulting in greater toxicity than predicted from the individual chemicals alone. The potential for synergistic interactions among mixtures of environmental contaminants raises significant concerns, as combined pollutants may have amplified harmful effects, emphasizing the importance of further research into these interactions [3,4,5,6,7]. Investigating the effects of concurrent exposure to multiple stressors is essential for accurately evaluating their ecological and physiological impacts on aquatic organisms. Understanding how agrochemicals and natural toxins interact within aquatic systems is therefore critical for developing more realistic environmental risk assessments and effective strategies for protecting ecosystem health.

Herbicides are widely used in modern agriculture to facilitate weed control and boost crop yields; however, their widespread use has led to unintended environmental consequences. Among these herbicides, Roundup^®^, which contains glyphosate [N-(phosphonomethyl) glycine] (GLY) as its active ingredient, is a leading global broad-spectrum herbicide [8]. GLY controls plant growth by inhibiting the shikimate metabolic pathway through 5-enoylpyruvylshikimate-3-phosphate synthase (EPSPS), an enzyme essential for producing aromatic amino acids critical to plant survival [9]. Although GLY has been widely used to control weed growth and enhance crop yields, its high water solubility and excessive use have raised concerns about environmental contamination, driving studies investigating its degradation, environmental persistence, and effects on non-target organisms, particularly aquatic species [9,10,11,12,13,14,15]. In addition to anthropogenic stressors, aquatic organisms can also be exposed to natural toxins such as microcystins (MCs) produced by cyanobacteria like *Microcystis aeruginosa* during harmful algal blooms (HABs) [16,17,18]. HABs can occur under conditions of elevated temperatures and nutrient enrichment, particularly nitrogen and phosphorus concentrations [17,19,20]. MCs with leucine and arginine (MC-LR) residues, are one of the most prevalent and toxic MCs variants found in freshwater HABs [21,22,23]. In aquatic environments, GLY-induced release of MCs from *Microcystis* may promote the co-occurrence of GLY and MC-LR, increasing the potential for simultaneous exposure in aquatic organisms [24]. Further, GLY and MC-LR can co-occur in agricultural freshwater ecosystems, where herbicide runoff and nutrient enrichment promote simultaneous exposure of aquatic organisms to both contaminants. Because GLY and MCs are typically quantified using different analytical methods, relatively few monitoring studies have measured both contaminants in the same water samples; however, several studies have documented their co-occurrence in agricultural watersheds [25,26,27,28,29].

Both GLY and MC-LR are recognized as hepatotoxic compounds that can adversely affect liver structure and function in exposed organisms [30,31,32]. The liver is the primary target organ for exogenous toxicants and plays a central role in numerous metabolic processes [33]. In aquatic vertebrates and invertebrates, GLY exposures led to genotoxicity, oxidative stress, inflammation, disruptions in antioxidant enzyme activity and lipid metabolism, and alterations in hepatic histopathology [34,35]. MC-LR damages liver cells by entering hepatocytes through Organic Anion Transporting Polypeptide (OATP) transport channels where it inhibits protein phosphatases (PP1 and PP2A), leading to oxidative stress, cytoskeletal disruption, and hepatocellular damage [36]. Numerous studies have demonstrated that both the natural toxin MC-LR and the anthropogenic contaminant GLY act as hepatotoxins in aquatic organisms, with hepatotoxic effects of MC-LR reported in the Chinese mitten crab (*Eriocheir sinensis*) [37], crab (*Neohelice granulata*) [38], and rusty crayfish (*Faxonius rusticus*) [39]. GLY-induced hepatotoxicity has been observed in common carp (*Cyprinus carpio*) [34] and grass carp (*Ctenopharyngodon idellus*) [40]. Although GLY and MC-LR may naturally co-occur, their combined toxic effects have been examined in a limited number of studies. For example, Zhang et al. [41] showed that co-exposure to GLY and MC-LR caused gill damage, increased oxidative stress, physiological alterations, and changes in gene expression in zebrafish (*Danio rerio*). Similarly, in zebrafish, Ding et al. [42] reported that both MC-LR and GLY induced intestinal damage and altered gut microbiota composition. Freshwater gastropods (*Lymnaea stagnalis*) exposed to both MC-LR and GLY showed increased accumulation in the digestive gland (hepatopancreas) and impaired catalase (CAT) and glutathione-S-transferase activity (GST) [43]. Additionally, a combined exposure altered the expression of proteins associated with oxidative stress pathways, biotransformation processes, and energy metabolism in mussels (*Unio pictorum*) [44]. Collectively, these findings suggest that co-exposure to MC-LR and GLY may produce enhanced or interactive toxic effects in aquatic organisms, highlighting the need for further investigation into their combined impacts on hepatic function and health.

Crayfish are widely used as model organisms in ecotoxicology and aquatic health studies due to their ecological importance, widespread distribution, and sensitivity to environmental contaminants [45,46]. As benthic invertebrates that inhabit sediments and occupy key trophic positions in freshwater food webs, crayfish are readily exposed to pollutants through the water column, sediments, and dietary intake, making them useful indicators of ecosystem health [47,48,49,50]. In addition, crayfish function as a keystone species by linking trophic levels and promoting energy flow within aquatic food webs [47,48,51]. Consequently, physiological analyses in crayfish provide valuable insight into the effects of environmental toxicants on aquatic organisms and environmental quality. In particular, the crayfish hepatopancreas (digestive gland) is an important target tissue for ecotoxicological assessment because it functions in digestion, nutrient storage, detoxification, biotransformation, and metabolism, analogous to the liver and pancreas in vertebrates [52,53]. The hepatopancreas accumulates contaminants and often exhibits measurable responses to pollutant exposure, including oxidative stress, altered enzyme activity, histopathological changes, cellular damage, and disruptions in metabolic processes [54,55,56,57,58,59]. This organ consists of numerous tubules separated by connective tissue and contains four epithelial cell types, including blister-like secretory cells (B cells), which possess large vacuoles involved in the sequestration and elimination of harmful substances [60]. Additionally, these large vacuolar structures have been identified as secondary lysosomes involved in the enzymatic degradation and biotransformation of xenobiotics and cellular autophagy [61]. Consequently, evaluating changes in hepatopancreatic morphology and cellular structure can serve as a sensitive biomarker of toxic pollutant exposure and provide evidence of contaminant-induced stress occurring in aquatic environments.

This study aimed to determine whether short-term exposure to environmentally relevant concentrations of Roundup^®^ (active ingredient GLY) and MC-LR, individually or in combination, induces morphological and physiological alterations in the hepatopancreas of *Procambarus acutus*, and whether combined exposure results in greater toxicity than exposure to either contaminant alone. Based on the environmental concentrations of MC-LR and GLY, we exposed crayfish to ethanol (control), 15 ppb MC-LR, 75 ppb GLY, and combination of both at the same concentrations (MC-LR + GLY) for seven days. Environmental concentrations of MC-LR range from 0 to 3000 ppb (µg L^−1^) [18], and GLY typically ranges from 0 to 887 ppb but has been recorded at concentrations above 5000 ppb [28,62,63,64,65]. Following all exposures, we collected hepatopancreas tissue for morphological and cellular analyses. We hypothesized that (1) individual exposure to MC-LR or GLY would induce hepatopancreatic tissue damage and reduce cell viability relative to controls, and (2) combined exposure would produce greater toxic effects than either contaminant alone because of their overlapping mechanisms of cellular injury, including oxidative stress and membrane damage. These hypotheses are supported by studies demonstrating synergistic effects of herbicide and cyanotoxin co-exposure in aquatic organisms; however, relatively few studies have examined the combined effects of MC-LR and herbicides [2,41,42,43,54]. The co-occurrence of GLY and MC-LR raises concerns regarding mixture toxicity, as simultaneous exposure to multiple contaminants may result in additive, synergistic, or otherwise altered biological effects compared with exposure to individual contaminants alone. To date, no studies have compared the morphological and physiological changes in the liver or hepatopancreas of aquatic organisms following combined MC-LR and GLY exposure with those observed following exposure to either contaminant alone. Understanding the effects of simultaneous exposure on the crayfish hepatopancreas is critical, as hepatopancreatic alterations may serve as indicators of impaired detoxification and biotransformation capacity and disrupted physiological function.

## 2. Materials and Methods

### 2.1. Crayfish Housing and Husbandry

Female White River crayfish (*P. acutus*) (weight: 14.56 ± 3.87 g, carapace length: 38.39 ± 3.48 mm, chelae length: 26.01 ± 3.25 mm; mean ± standard deviation (S.D.), *N* = 24), used in this experiment were obtained from Toledo Goldfish (Stoutland, MO, USA). All crayfish were housed in tanks at the University of Detroit Mercy for at least 2 weeks before experimentation [temperature = 22.3 °C; 14:10 h light dark cycle]. During this time and throughout the exposure period, crayfish were fed one rabbit pellet per crayfish three times per week.

### 2.2. Experimental Treatments

#### 2.2.1. Preparation and Standardization of MC-LR and GLY Solutions

Water, methanol, ethanol, acetonitrile, and formic acid were purchased from MilliporeSigma (St. Louis, MO, USA) and were LC-MS grade except as noted.

An initial stock solution of MC-LR was prepared by dissolving MC-LR (Focus Biomolecules (Plymouth Meeting, PA, USA); 95% purity) in ethanol [54]. A 3-ppm stock solution of MC-LR was prepared by dilution with deionized water. The MC-LR treatment solutions were prepared by diluting the 3-ppm stock solution with dechlorinated water to a final concentration of 15 ppb (0.006% ethanol). A 15-ppm GLY stock solution was prepared by diluting Roundup^®^ Pro Concentrate (Bayer (Leverkusen, North Rhine-Westphalia, Germany); 445 g L^−1^ GLY) in ethanol [66]. The GLY exposure solutions were prepared by further dilution with dechlorinated water to 75 ppb. For the combined MC-LR + GLY treatment, 5 mL of 3-ppm stock MC-LR and 5 mL of 15-ppm stock GLY were added to 990 mL dechlorinated water in treatment tanks. To control for exposure to ethanol in treatment groups, control animals were exposed to final concentration of 0.006% ethanol. The control solutions were prepared by diluting 0.6% ethanol with dechlorinated water.

All exposure solutions were verified and standardized via liquid chromatography–mass spectrometry (LC-MS) analysis (see Table S1). For preparation of MC-LR standards, a 2.5 mg mL^−1^ solution of MC-LR (MilliporeSigma; ≥95% purity) in DMSO was first diluted to 10,000 ppb in water and subsequently diluted to 1000 ppb with additional water. Standards of 0, 3, 10, 20, and 30 ppb were prepared by diluting the 1000 ppb stock solution in water. The MC-LR exposure solutions were analyzed by reversed phase LC-MS using an Agilent 1290 UHPLC coupled to an Agilent 6220 Accurate-Mass Time-of-Flight Mass Spectrometer (Santa Clara, CA, USA). The column used was a Phenomenex Kinetex 2.6 μm C18 column (2.1 × 75 mm) (Torrance, CA, USA). Mobile phase A was 0.1% formic acid in water, and mobile phase B was 0.1% formic acid in acetonitrile. The LC flow rate was 0.2 mL min^−1^, and the gradient consisted of a 3-min linear ramp from 10 to 90% B, a 1-min wash at 90% B, and a 4-min re-equilibration period at 10% B (total run time 8 min). The injection volume was 20.0 μL, and the column temperature was 30 °C. Accurate mass full-scan (*m*/*z* 50–1200) detection was performed in positive-ion mode, monitoring for the *m*/*z* 995.5566. Features were quantitated using MassHunter Quantitative Analysis software. The limit of detection (LOD) and limit of quantitation (LOQ) for MC-LR were calculated according to the ICH Q2(R1) guideline as 3.3 σ/S and 10 σ/S, respectively, where σ is the standard deviation of the peak area obtained from replicate analyses of the lowest concentration standard and S is the slope of the calibration curve [67]. The LOD and LOQ for MC-LR were 0.6 ppb and 2 ppb, respectively.

To standardize GLY solutions, a 1-mg mL^−1^ stock solution of GLY (MilliporeSigma (St. Louis, MO, USA); PESTANAL^®^ analytical standard grade) was prepared by dissolving the solid in water. The GLY stock was first diluted to 10,000 ppb in water and then subsequently diluted to 1000 ppb with additional water. A 1-mg mL^−1^ stock solution of the internal standard, GLY-d_2_ (Cayman Chemical (Ann Arbor, MI, USA): ≥98% purity), was prepared by dissolving the solid in DMSO. The GLY-d_2_ stock was first diluted to 10,000 ppb in water and then subsequently diluted to 1000 ppb with additional water. Glyphosate standards of 0, 3, 10, 20, 30, and 100 ppb, each with 10 ppb GLY-d_2_, were prepared in water. All glyphosate exposure solutions and standards were derivatized for reversed-phase liquid chromatography-tandem mass spectrometry analysis using a method reported elsewhere [68]. Briefly, 0.5 mL of each solution was mixed with 0.5 mL of borate buffer solution (50 g L^−1^, pH = 9). The mixture was vortexed, and 0.5 mL of 9-fluorenylmethylchloroformate (FMOC) reagent was added. The solution was gently swirled, and the derivatization reaction was completed at 50 °C water bath for 20 min. The samples were cooled and diluted 50% with methanol for LC-MS analysis. Derivatized solutions were analyzed on an Agilent (Santa Clara, CA, USA) 1290 Binary pump coupled to an Agilent 6490 triple quadrupole instrument equipped with a Jetstream electrospray ionization source. Chromatographic separation was performed using a Waters Acquity HSS T3 1.8 µm column, 100 mm × 2.1 mm ID (Waters Corporation, Milford, MA, USA). Mobile phase A was 100% water with 0.1% formic acid and mobile phase B was 100% methanol with 0.1% formic acid. The gradient was as follows: 0–5 min 5–100% B, hold 99% B for 2 min, return to 0% B at 7.1 min; total run time 10 min. The flow rate was 0.45 mL min^−1^ and the column temperature was 55 °C. The injection volume was 5 µL. Mass spectrometry was performed by electrospray ionization in negative ion multiple reaction monitoring (MRM) mode. Source parameters were: drying gas temperature 275 °C, drying gas flow rate 15 L min^−1^, nebulizer pressure 35 psig, sheath gas temp 250 °C and flow 11 L min^−1^, capillary voltage −3500 V, nozzle voltage −2000 V. Ion funnel parameters were: high pressure RF −90 V and low pressure RF −60 V. MRM transitions parameters for GLY were: precursor ion *m*/*z* 390.1, MS1 res unit: product ion *m*/*z* 168.0, MS2 res unit: dwell 100 msec, fragmentor 380, collision energy 18, cell accelerator voltage 7. GLY-d_2_ was analyzed using the same parameters except the precursor ion was *m*/*z* 392.1 and the product ion was *m*/*z* 170.0. Delta EMV (–) was set at 600. Features were quantitated by peak area relative to internal standard using MassHunter Quantitative Analysis software. The LOD and LOQ for GLY were calculated according to the ICH Q2(R1) guideline as 3.3 σ/S and 10 σ/S, respectively, where σ is the standard deviation of the relative peak area obtained from replicate analyses of the lowest concentration standard and S is the slope of the calibration curve [67]. The LOD and LOQ for GLY were 0.8 ppb and 2 ppb, respectively.

#### 2.2.2. Exposure to MC-LR and GLY

In this experiment, crayfish were exposed to MC-LR (15 ppb), GLY (75 ppb), and a combination of both MC-LR (15 ppb) + GLY (75 ppb) for 7 days. This was done by individually isolating female crayfish in 1.5-L translucent plastic containers with lids (19 cm diameter and 9.5 cm depth), each of which was filled with 500 mL of dechlorinated water and individually aerated with an air stone. On exposure days 0, 2, 4, and 6, crayfish were administered MC-LR, GLY, or a combined MC-LR + GLY treatment [55]. On day 7, crayfish were rinsed, anesthetized by cooling in a −20 °C freezer, weighed, and dissected for hepatopancreas collection. Hepatopancreatic tissues were subsequently processed for histological and flow cytometric analyses to evaluate treatment-induced morphological and cellular alterations.

### 2.3. Tissue Collection, Processing, Staining, and Analysis

Following dissection of the hepatopancreas, the tissue was placed in 4% paraformaldehyde for at least 48 h. Hepatopancreas tissues were then embedded in paraffin using a standard embedding procedure (see Hadeed et al. [55]). Briefly, tissues were dehydrated in an ascending alcohol series (15 min changes each of 50%, 75%, 95%, and 100% ethanol, three replicates each), cleared in xylene (15 min, three replicates each), and infused with paraffin (70 °C; 15 min, three replicates each), and cooled in a tissue mold. Tissue blocks were sectioned (5 µm) using a microtome (Microm HM 325A, Waldorf, Germany), collected on slides, and dried on a slide warmer. Hematoxylin and eosin (H&E) staining was performed to assess hepatopancreas morphology, following a protocol adapted from Bancroft and Gamble [69]. Tissue sections were first deparaffinized using xylene (1-min, three replicates each) and then gradually rehydrated through a descending ethanol series. Hematoxylin was applied for 5 min to stain cell nuclei, followed by a brief decolorization step. Slides were then counterstained with eosin for 10 min to highlight cytoplasmic structures. After staining, tissues were dehydrated sequentially with 95% ethanol (1-min, single replicate) and 100% ethanol (1-min, three replicates). Samples were cleared with xylene (1-min, three replicates) and cover slipped using Permount mounting medium. Prepared slides were imaged using a Nikon Eclipse light microscope equipped with a DAGE-MTI color camera (Tokyo, Japan).

H&E-stained sections were evaluated for histopathological alterations in the hepatopancreas, including changes in tubular epithelium, vacuolization, and tubule morphology, and these features were compared between control and treated crayfish [55,70,71]. For each morphological parameter measured, Fiji was used to analyze 20 representative hepatopancreatic tubules per crayfish, yielding a total of 100 tubules examined for each treatment group [72]. The number of vacuoles per tubule was quantified, with coalesced vacuoles forming a single larger vacuole counted as one vacuole [54,55,73]. All vacuole counts were performed using the Cell Counter plugin (K. De Vos, University of Sheffield) in Fiji. The proportion of each tubule occupied by vacuoles (vacuole proportion) was quantified using the Fiji Color Counter plugin (Wayne Rasband, ImageJ, version 1.54p). All vacuoles within each tubule were manually outlined in Fiji, and the total vacuole area was measured as the number of pixels occupied by vacuoles. The vacuole proportion was then calculated by dividing the total vacuole pixel count by the total number of pixels within the tubule epithelium, yielding the proportion of the epithelium occupied by vacuoles. Epithelial height was quantified by measuring the distance from the basal to the apical surface at four equidistant points along each tubule and averaging the measurements [54,55]. To examine epithelial degeneration, tubule lumen proportion was measured using the Fiji Color Counter plugin [54,74]. Pixel counts were determined for the entire tubule and for the lumen alone. Lumen proportion was calculated as the ratio of lumen pixels to total tubule pixels for each tubule across all exposure treatments.

### 2.4. Flow Cytometry Analysis

Hepatopancreas tissues were also examined for cellular changes using flow cytometry. The excised hepatopancreas was weighed, and the tissue was divided into 0.35 g portions. Each 0.35 g portion of hepatopancreatic tissue was placed in a petri dish and resuspended in phosphate-buffered saline (PBS; 137 mM NaCl, 2.7 mM KCl, 10 mM Na_2_HPO_4_, 2 mM KH_2_PO_4_) supplemented with 5% fetal bovine serum and 5.5 mM ethylenediaminetetraacetic acid (EDTA). The ratio of PBS to tissue was maintained at 1 mL per 0.35 g of tissue [75]. The tissue was gently homogenized using a manual homogenizer by pressing it against the Petri dish. The resulting cell suspension was filtered through a 100-µm mesh filter to remove large debris. Cells were resuspended in 500 µL PBS and centrifuged for 10 min at 500× *g* at 4 °C. The supernatant was removed by vacuum suction, and the pellet was stained.

To assess cell viability, cell suspensions were stained separately with fluorescein diacetate (FDA) and propidium iodide (PI) [39,75]. FDA was used to identify metabolically active cells, which fluoresce green following intracellular enzymatic conversion, whereas PI, a membrane-impermeable dye, fluoresces red in cells with compromised membrane integrity, indicating nonviable cells [76]. To prepare the stains, a 1 mg mL^−1^ acetone stock of FDA (Sigma Aldrich, St. Louis, MO, USA) and 1 mg mL^−1^ stock of PI (Sigma Aldrich, St. Louis, MO, USA) were diluted to a final concentration of 1:10,000 in 0.1 M PBS for a final volume of 1 mL of each stain mixture to each of the cell suspensions. Additionally, blank samples were prepared using the same protocol but without the addition of fluorescent stains; an equivalent volume of PBS was added instead. Samples were analyzed by flow cytometry using a CytoFLEX LS flow cytometer (Beckman Coulter, Brea, CA, USA) equipped with 488-nm and 633-nm lasers. Data acquisition was performed using CytExpert software. Analyses were executed on the scattering signals (forward scatter and side scatter) as well as on channels FL1 (green fluorescence, 530/30) and FL3 (red fluorescence, 670 Long Pass). All flow cytometry analyses were conducted at the University of Detroit Mercy School of Dentistry (Detroit, MI, USA). A total of 48 samples were analyzed by flow cytometry, comprising 24 FDA-stained samples and 24 PI-stained samples. Each treatment group included six biological replicates for each staining assay (*n* = 6 per treatment) and each sample was analyzed in duplicate, and the resulting values were averaged. Flow cytometry data was collected using Cytexpert version 2.7 (Beckman Coulter). An average of 4598 ± 508 events/sample were collected. Cell debris and autofluorescent events were excluded by freehand gating and cell populations were identified and analyzed using forward scatter (FSC) and side scatter (SSC) characteristics (Figure S1).

### 2.5. Statistical Analysis

Statistical analyses were run in R statistical software version 4.6.0 [77] and RStudio version 2026.05.0 [78]. Outliers were checked for each variable using visual inspection via dot plots constructed in R in addition to using the detect outliers’ function in the rstatix package [79], which defines outliers based on 1.5× interquartile range below the first quantile and above the third quantile. Outliers were detected but were not removed.

Differences in morphological variables (vacuole number, vacuole proportion, lumen proportion, epithelial height) were evaluated using generalized linear models (GLMs) with different link functions appropriate to the distribution of the data. In all models, individual was included as a random effect, with treatment as the main effect. Vacuole number was analyzed using a GLM with a negative binomial link function, which is appropriate for count data, implemented in the R package lme4 [80]. Vacuole and lumen proportion were analyzed using a GLM with a beta regression link function, which is appropriate for proportion data, implemented in the R package glmmTMB [81]. Epithelial height was analyzed using a linear mixed effects model, which accommodates normally distributed variables such as this, implemented using the package nlme [82]. All models were summarized using Type II sums of squares using the Anova function in the R package car [83]. Differences between treatments were evaluated using Tukey posthoc tests implemented in the R package emmeans [84]. All model assumptions were reasonably met for these data. Please refer to the analysis files included in the repository for additional details.

Flow cytometry data were analyzed using linear mixed effects models implemented in the package nlme. Before analysis, both cell count variables were transformed via Box-Cox transformation in order to align with the assumption of normally distributed data for the model. The box-cox transformation was chosen based on analysis using the bestNormalize package. Each model had individual as a random effect with treatment as the main effect. All models were summarized using Type II sums of squares using the Anova function in the R package car [83]. Differences between treatments were evaluated using Tukey posthoc tests implemented in the R package emmeans [84]. All model assumptions were reasonably met for these data. Please refer to the analysis files included in the Data Availability section for additional details. All figures were generated using GraphPad Prism (version 10.4.0).

## 3. Results

### 3.1. Histological Analysis

**Figure 1:**
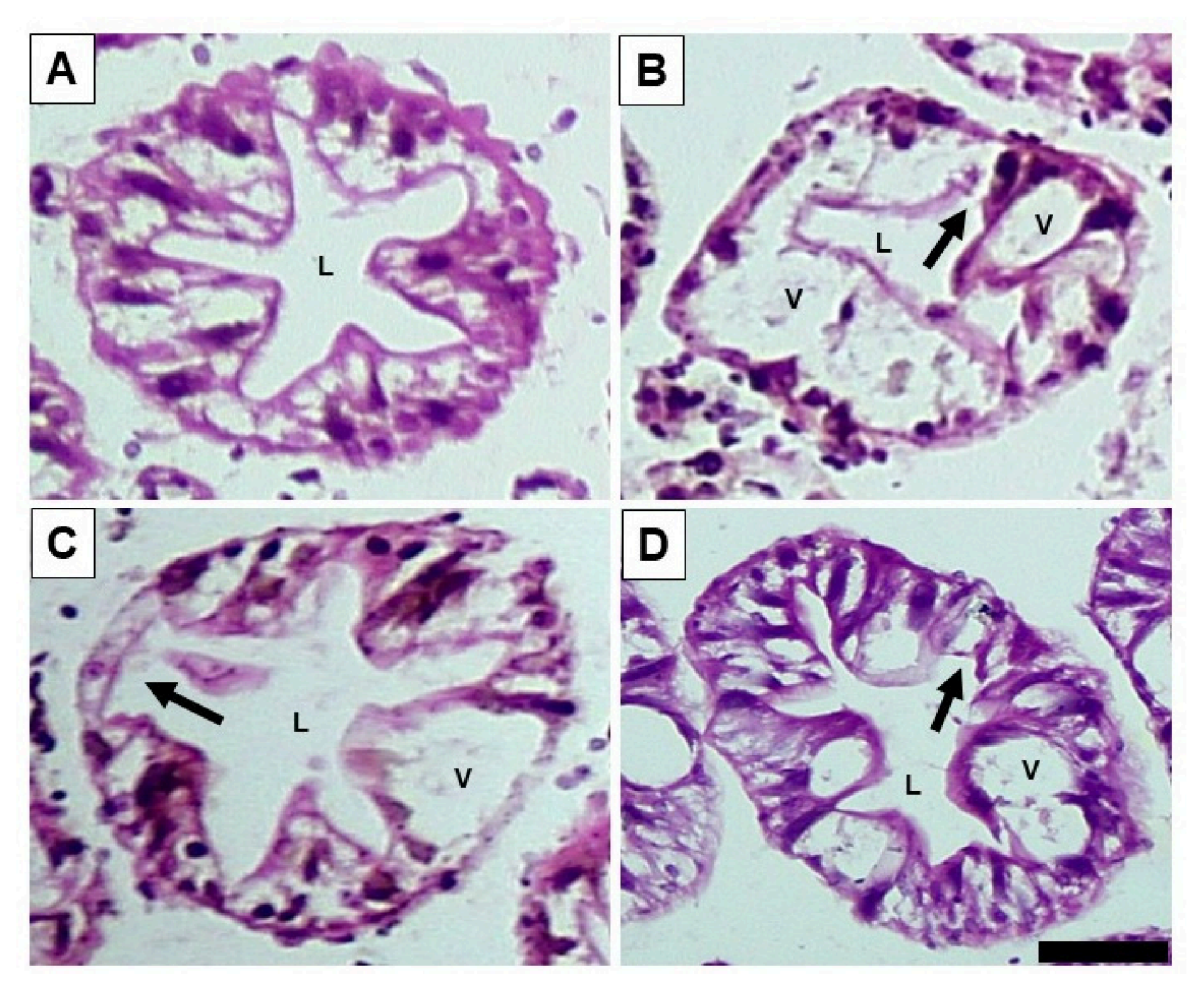
Representative tubule cross sections from the hepatopancreas of control (**A**), MC-LR (**B**), GLY (**C**), and MC-LR + GLY-treated (**D**) crayfish. Control tubules displayed typical asterisk-shaped lumen with intact microvillar brush borders, while treatment groups displayed epithelial degeneration (arrows). Vacuolization (v) and tubule lumen (L) dilation were noted in some treatment groups. Scale bar is 50 µm.

Tubule morphology was altered following exposure to MC-LR, GLY, and MC-LR + GLY (Figure 1). Hepatopancreatic tubules from control crayfish exhibited a well-organized, continuous, and uniform architecture, characterized by distinct asterisk-shaped lumens. An intact microvillar brush border and a narrow central lumen was observed throughout all examined sections. Numerous small lipid vacuoles were present within the tubules, whereas large vacuoles were restricted to a small number of B cells (Figure 1A). This morphology is consistent with typical hepatopancreatic histological morphology previously reported by Desouky et al. [70] and serves as a baseline for comparison with the effects of MC-LR, GLY, and their combination. Compared with controls, hepatopancreatic tubules from crayfish exposed to MC-LR, GLY, and MC-LR + GLY exhibited varying degrees of microvillar brush border disruption, an increased number of B cells containing large vacuoles, and lumen dilation (Figure 1B–D).

**Figure 2:**
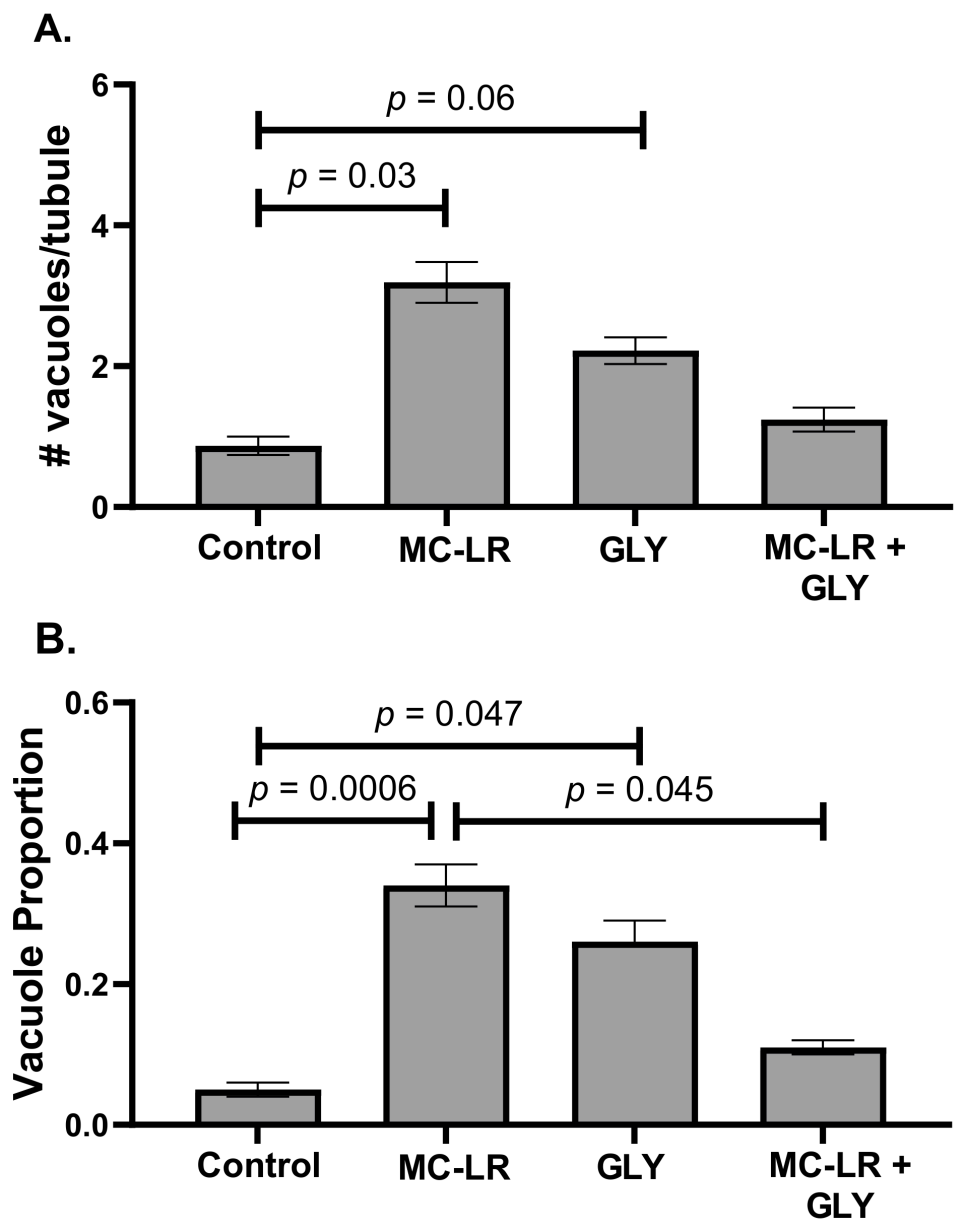
Quantification of hepatopancreatic tubule vacuolization in control, MC-LR-, GLY-, and MC-LR + GLY-exposed crayfish. (**A**) The mean number of vacuoles per tubule was compared among treatment groups. MC-LR-exposed crayfish exhibited significantly more vacuoles per tubule than controls, while GLY-exposed crayfish showed a marginally significant increase in vacuole number relative to controls. (**B**) The proportion of the tubular epithelium occupied by vacuoles was also assessed. Crayfish exposed to MC-LR and GLY displayed a significantly greater vacuolated proportion of the tubule compared with controls. In addition, MC-LR-exposed crayfish exhibited a significantly greater proportion of tubule occupied by vacuoles than GLY-exposed crayfish.

Quantitative analysis of hepatopancreas morphology showed differences across treatments in several morphological measurements. Vacuole number showed a significant difference across treatment groups (χ^2^ _(3, *n* = 400)_ = 11.439, *p* = 0.01; Figure 2A). Posthoc Tukey contrasts showed a significant increase in vacuole number between control (mean ± standard error (S.E.)) (0.87 ± 0.13) and MC-LR-treated animals (3.19 ± 0.29; *p* = 0.03), corresponding to a 3.7-fold increase relative to controls. A marginal increase was observed between control and GLY-treated animals (2.22 ± 0.19; *p* = 0.06), corresponding to a 2.6-fold increase relative to controls. No differences were detected between control and MC-LR + GLY-treated animals (1.24 ± 0.17; *p* > 0.05) nor between any of the treatment groups. The proportion of the hepatopancreas taken up by vacuole(s) (hereafter, vacuole proportion) was also found to differ significantly across treatment groups (χ^2^ _(3, *n* = 400)_ = 16.659, *p* = 0.0008; Figure 2B). Posthoc Tukey contrasts showed that, compared to control (0.05 ± 0.01), vacuole proportion was significantly greater in GLY-treated animals (0.26 ± 0.03; *p* = 0.047), representing a 6.8-fold increase relative to controls. MC-LR-treated animals also exhibited a significant increase in vacuole proportion compared with controls (0.34 ± 0.3; *p* = 0.0006), corresponding to a 5.2-fold increase. No significant differences were observed between control and MC-LR + GLY-treated animals (0.11 ± 0.02; *p* > 0.05) nor between any of the treatment groups (*p* > 0.05).

**Figure 3:**
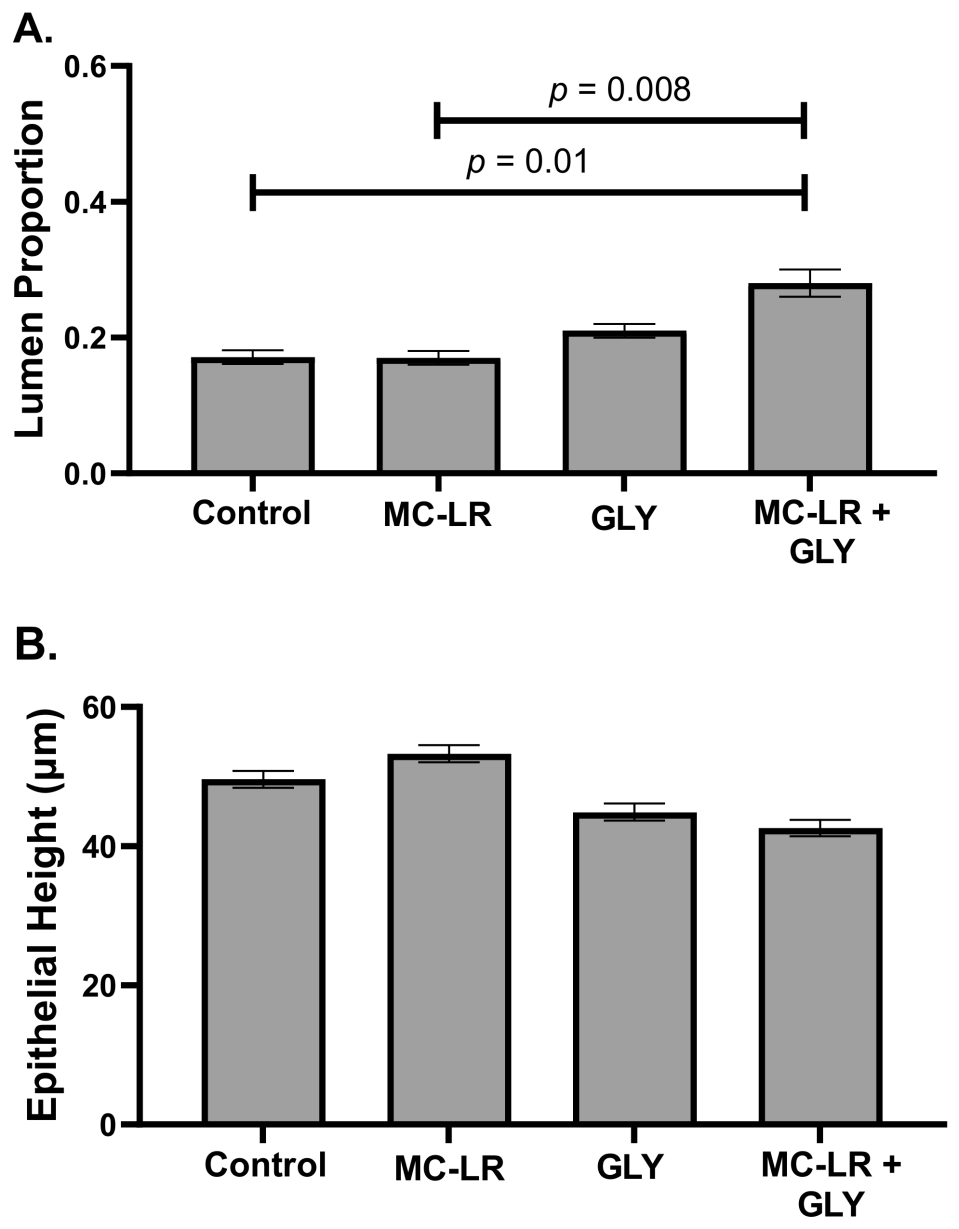
Analysis of hepatopancreatic tubule lumen proportion and epithelial height in control, MC-LR-, GLY-, and MC-LR + GLY-exposed crayfish. Crayfish exposed to the combined MC-LR + GLY treatment exhibited a significantly greater lumen proportion compared with both control and MC-LR-exposed animals (**A**). No significant differences in tubule epithelial height were observed among treatment groups (**B**).

The proportion of the hepatopancreas taken up by the lumen (hereafter, lumen proportion) also significantly differed among treatments groups (χ^2^ _(3, *n* = 400)_ = 13.318, *p* = 0.004; Figure 3A). Posthoc Tukey contrasts showed that, compared to control (0.17 ± 0.01), lumen proportion was significantly greater in crayfish treated with MC-LR + GLY (0.28 ± 0.02; *p* = 0.01), representing an approximately 65% increase relative to controls. Lumen proportion did not significantly differ in MC-LR (0.17 ± 0.01; *p* > 0.05) or GLY-treated animals (0.21 ± 0.01; *p* > 0.05). However, we did find a significantly greater lumen proportion in MC-LR + GLY-treated animals compared to MC-LR-treated animals (*p* = 0.008), but there were no differences between other treatment groups. Conversely, epithelial height did not significantly differ among treatments (χ^2^ _(3, *n* = 400)_ = 4.759, *p* = 0.19; Figure 3B). Epithelial heights were 49.63 ± 1.29 µm, 53.33 ± 1.21 µm, 44.88 ± 1.24 µm, and 42.60 ± 1.17 µm for control, MC-LR, GLY, and MC-LR + GLY treated groups, respectively.

### 3.2. Flow Cytometry

**Figure 4:**
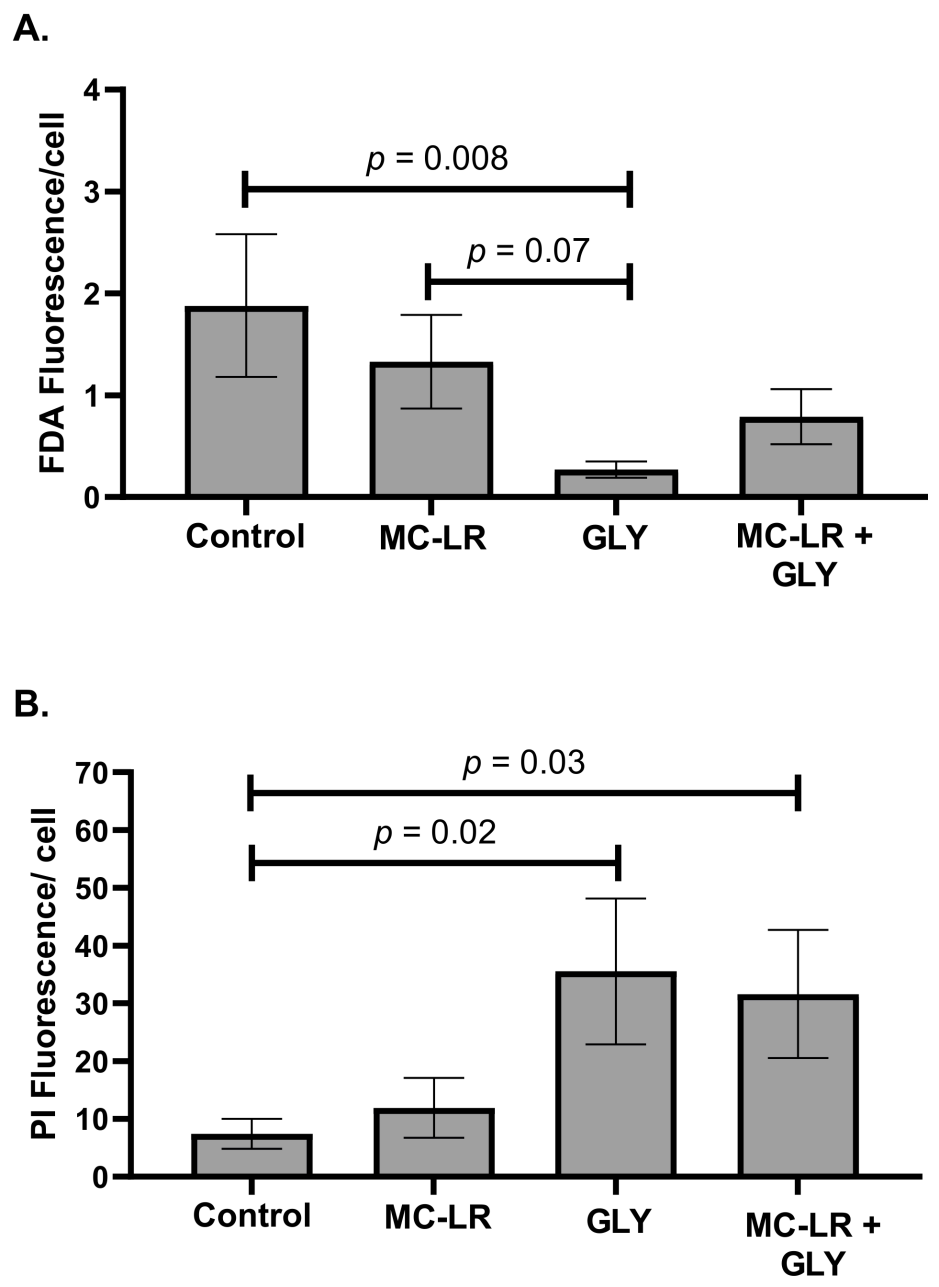
Assessment of hepatopancreatic cell viability and membrane integrity following exposure to MC-LR, GLY, and their combination (MC-LR + GLY) using FDA and PI staining. FDA-positive cells, indicative of viable cells, were significantly reduced in GLY-exposed crayfish compared with controls and were marginally lower than in MC-LR-exposed crayfish (**A**). PI-positive cells, indicative of compromised membrane integrity, were significantly increased in both the GLY and MC-LR + GLY treatment groups relative to controls (**B**).

The blanks, prepared without any dyes, exhibited minimal background fluorescence, ensuring accurate discrimination between stained cell populations. Overall, we found that treatment groups significantly differed in the number of FDA- (χ^2^ _(3, *n* = 6)_ = 14.287, *p* = 0.003; Figure 4A) and PI-positive cells (χ^2^ _(3, *n* = 6)_ = 14.633, *p* = 0.002) in the hepatopancreas. Posthoc Tukey contrasts revealed that the number of viable (FDA-positive) cells, compared to control (mean ± S.E. = 1.88 ± 0.50), was significantly lower in GLY-treated animals (0.27 ± 0.06; *p* = 0.008), representing an approximately 86% reduction in viable cells. No significant differences were observed in the MC-LR-treated (1.33 ± 0.31; *p* > 0.05) or MC-LR + GLY-treated (0.79 ± 0.19; *p* > 0.05) animals compared to controls. A marginal reduction was observed in the number of FDA-positive cells in the GLY-treated animals compared to MC-LR-treated animals (*p* = 0.07). No other differences were detected among treatment groups. Posthoc Tukey contrasts showed that the number of non-viable (PI-positive) cells, compared to control (7.40 ± 1.80), was significantly higher in GLY (35.53 ± 8.64; *p* = 0.02) and MC-LR + GLY-treated (31.58 ± 7.74; *p* = 0.03) animals, corresponding to 4.8-fold and 4.3-fold increases, respectively. PI-positive cell numbers did not differ between MC-LR-treated (11.92 ± 4.00; *p* > 0.05) animals and controls. No differences were detected in the number of PI-positive cells among treatment groups (Figure 4B). Overall, MC-LR exposure alone did not significantly alter cell viability or membrane integrity.

## 4. Discussion

This study demonstrated that exposure to MC-LR, GLY, and their combined treatment (MC-LR + GLY) altered hepatopancreatic tubule morphology in crayfish (Figure 1). Given the central role of the hepatopancreas in xenobiotic detoxification and biotransformation, digestion, and nutrient storage, structural alterations within this organ may have detrimental consequences for organismal health and physiological function [85]. Accordingly, hepatopancreatic morphology is widely used as a sensitive indicator of toxicant-induced stress and tissue injury in crayfish [46,52]. Consequently, alterations to hepatopancreatic structure may have significant physiological implications, potentially impairing metabolic function, nutrient processing, and the organism’s capacity to detoxify and respond to environmental contaminants. We found that individual exposure to MC-LR and GLY increased vacuolization, as evidenced by greater vacuole number and/or vacuole proportion (Figure 2), which may reflect increased sequestration of xenobiotics [52,54,60,86]. The combined MC-LR + GLY treatment produced the greatest increase in tubule lumen proportion, which may reflect dilation of the hepatopancreatic tubules and degeneration of the tubule epithelium (Figure 3A). However, epithelial height remained unchanged across all treatments (Figure 3B). At the cellular level, hepatopancreatic cell integrity was adversely affected by exposure, as indicated by a reduction in viable, FDA-positive cells following GLY treatment and an increase in membrane-compromised, PI-positive cells in both the GLY and MC-LR + GLY treatment groups (Figure 4). Notably, combined exposure did not consistently produce greater effects than those observed following exposure to the individual toxicants. The results suggest that MC-LR and GLY exert distinct, complementary toxicological effects on the crayfish hepatopancreas. Although co-exposure altered tubule morphology by increasing lumen dilation, the mixture did not consistently produce responses greater than those observed for the individual toxicants across all endpoints. Consequently, the data do not provide sufficient evidence to classify the interaction as synergistic or antagonistic, but rather indicate endpoint-specific combined effects that warrant further investigation using formal mixture toxicity approaches [4].

Histopathological examination following exposures to MC-LR and GLY and their combination revealed brush border disruption, suggesting impaired hepatopancreatic integrity and function. [85]. MC-LR exposure has been reported to disrupt hepatopancreatic tubular architecture, including damage to the microvillar brush border, in several crustacean species, including the giant freshwater prawn (*Macrobrachium rosenbergii*) [87], crayfish (*Faxonius virilis*) [54], and white shrimp (*Litopenaeus vannamei*) [88,89]. Similarly, GLY exposure caused structural changes in the hepatopancreas of the prawn (*M. potiuna*) [66]. These GLY-induced structural changes may be caused by hypoxic conditions created in tissues following exposure [90]. In addition to tubular damage, increased vacuolization and enlarged B-cell vacuoles suggest hepatopancreatic uptake of MC-LR and GLY, as B-cells absorb luminal contents through endocytosis [52,91]. Increased vacuolization occurred in crayfish exposed to MC-LR or GLY individually, whereas no increase was observed following co-exposure. This finding suggests that combined exposure to MC-LR and GLY may alter cellular processes associated with vacuole formation, resulting in a response distinct from that observed following individual exposures. These histopathological alterations demonstrate that both MC-LR and GLY compromise hepatopancreatic structure and function, while co-exposure produces a distinct tissue response that may reflect complex interactions between these contaminants.

Degeneration of the tubular epithelium and subsequent loss of tubule integrity may also contribute to lumen dilation. Consistent with this interpretation, co-exposure to MC-LR and GLY resulted in significant enlargement of the tubule lumen. Comparable histopathological changes have been reported in crustaceans exposed to MCs, pesticides, herbicides, and other aquatic contaminants, suggesting that these responses are common indicators of toxicant-induced stress [54,55,71,92,93]. These changes are likely caused by increased oxidative stress, which can damage epithelial cells and microvilli, disrupt cellular homeostasis, and impair nutrient processing and storage functions within the hepatopancreas [46]. Given that both MC-LR and GLY are known to induce oxidative stress and lipid peroxidation in crustaceans, these mechanisms may have contributed to the observed effects, although they were not directly measured in this study [37,38,88,92,93]. To date, only one other study has investigated the combined effects of MCs and GLY on the hepatopancreas of an aquatic invertebrate. Lance et al. [43] examined the effects of MC-producing cyanobacteria (33 µg L^−1^) and Roundup^®^ (GLY concentration: 1 µg L^−1^) on the freshwater gastropod (*L. stagnalis*). Following a 21-day exposure, they showed that co-exposure increased CAT activity, indicating activation of antioxidant defense mechanisms; however, CAT activity in the combined treatment was not elevated beyond levels observed following exposure to either MCs or GLY alone. These results suggest that co-exposure did not elicit a synergistic oxidative stress response under the experimental conditions. Consistent with these findings, our results support the growing body of evidence that both MC-LR and GLY exposures contribute to hepatopancreatic damage; however, a co-exposure to both MC-LR and GLY did not lead to increased overall hepatopancreas damage, as hypothesized. Collectively, the histopathological alterations observed following exposure to MC-LR and GLY, including changes in tissue architecture and cellular morphology, indicate disruption of hepatopancreatic structure and suggest compromised tissue function and cellular integrity.

Flow cytometric analysis of hepatopancreas cells revealed that GLY exposure significantly reduced the proportion of FDA-positive cells, indicating decreased cellular viability and metabolic activity. In addition, both GLY and the combined MC-LR + GLY treatment significantly increased the proportion of PI-positive cells, demonstrating compromised plasma membrane integrity. In contrast, exposure to MC-LR alone did not significantly affect either endpoint, indicating that GLY exposure had a greater negative impact on cell viability and plasma membrane integrity under the conditions examined. Two studies [41,42] reported that a 21-day exposure to MC-LR (35 μg L^−1^) and GLY (3.5 mg L^−1^) in zebrafish induced inflammation, oxidative stress, and lipid peroxidation in gill and intestinal tissues; however, the combined exposure did not produce additive effects on these responses. Further, exposing mussels (*U. pictorum*) to both MC-LR (10 μg L^−1^) and GLY (10 μg L^−1^) for seven days disrupted the expression of proteins associated with oxidative pathways, detoxification processes, and energy metabolism in the digestive gland [44]. Thus, the reduction in viable cells and increase in membrane-compromised cells following GLY exposure may result from oxidative stress, mitochondrial dysfunction, and membrane lipid peroxidation, all of which have been reported as mechanisms of GLY-induced toxicity. The comparatively weaker effects observed following MC-LR exposure may reflect a concentration or exposure length below the threshold required to induce measurable cytotoxicity, the occurrence of histopathological alterations prior to extensive cell death, or the presence of detoxification mechanisms that mitigate acute cytotoxic effects in crayfish. These flow cytometric findings are consistent with the histological observations of tissue damage and structural disruption in the hepatopancreas, suggesting that the observed lesions are associated with reduced cellular integrity. Collectively, the results indicate that GLY exerts stronger effects on hepatopancreatic cell viability and membrane integrity than MC-LR under the exposure conditions examined in this study, despite both contaminants inducing histopathological alterations.

Overall, the findings of this study have important ecological implications because cyanobacterial toxins (e.g., MC-LR) and GLY can co-occur in freshwater ecosystems, particularly in agricultural watersheds where nutrient runoff promotes harmful algal blooms and herbicide contamination [24]. Furthermore, MC-LR and GLY often co-occur with a variety of other contaminants in aquatic environments, making it essential to understand their combined effects and the potential risks posed by exposure to complex contaminant mixtures [28,62,94]. As benthic organisms that inhabit sediments and forage on a diverse range of food sources, crayfish are likely to experience chronic exposure to contaminant mixtures in natural environments, with these contaminants having the potential to accumulate within their tissues [45]. The histopathological and cellular alterations observed in the hepatopancreas following exposure to MC-LR, GLY, and their combination, including brush border disruption, tubule dilation, vacuolization, and reduced cell viability, suggest impairments in digestion, nutrient absorption, and energy storage. Such physiological disruptions may reduce growth, reproductive success, and overall fitness while increasing susceptibility to additional environmental stressors such as disease, hypoxia, temperature fluctuations, and predation. Like other studies in fish and aquatic invertebrates [33,34,35,36], this study did not show significant additive effects of MC-LR and GLY in the hepatopancreas; however, understanding their combined toxicity remains important, as additive or synergistic effects may occur in other tissues, at different exposure concentrations or durations, or when these contaminants interact with additional environmental stressors and pollutants commonly present in aquatic ecosystems. For example, Banaee et al. [95] reported that co-exposure to GLY and the insecticide chlorpyrifos produced synergistic effects on biochemical markers of oxidative stress in the hemolymph of the crayfish (*Pontastacus leptodactylus*). A limitation of the present study is that only a single environmentally relevant concentration of MC-LR and GLY was evaluated. Consequently, although the study provides insight into the effects of combined exposure, it was not designed to determine whether the interaction between these contaminants is antagonistic, additive, or synergistic using formal mixture toxicity approaches such as concentration addition, independent action, isobologram analysis, or combination index analysis [4]. Because crayfish are considered keystone species and ecosystem engineers in many freshwater ecosystems [47,48,51,96], declines in their health and performance could have cascading effects on benthic food webs, altering predator–prey interactions, organic matter processing, nutrient cycling, and the trophic transfer of energy and contaminants through aquatic food webs. As an important prey item for fish, birds, reptiles, and mammals, crayfish can also serve as a pathway for the transfer of accumulated contaminants to higher trophic levels [97,98]. Consequently, contaminant-induced damage to crayfish may extend beyond individual-level effects and contribute to broader changes in ecosystem structure, contaminant dynamics, and ecosystem functioning. Given the ecological significance of crayfish in freshwater systems, co-exposure to these stressors may influence community structure and aquatic ecosystem health.

## 5. Conclusions

This study demonstrates that exposure to the environmental contaminants MC-LR and GLY (Roundup^®^), both individually and in combination, induces histopathological and cellular alterations in the hepatopancreas of *P. acutus*. MC-LR primarily increased vacuolization, indicating cellular stress and altered metabolic function, whereas GLY had more pronounced effects on hepatopancreatic cell viability and membrane integrity, as shown by reduced viable cells and increased membrane-compromised cells. Combined exposure produced distinct pathological responses, particularly increased tubule lumen dilation and membrane damage, but did not consistently exacerbate all endpoints compared to single exposures, suggesting largely non-additive interactions between these contaminants. These findings are consistent with the idea that organisms may generate complex, endpoint-specific response profiles when exposed to contaminant mixtures rather than simple additive effects, potentially due to differences in toxicological targets, uptake pathways, or activation of detoxification mechanisms [41]. Although no consistent synergism was observed in the hepatopancreas under the conditions tested, interactions may differ across tissues, exposure durations, concentrations, or in the presence of additional environmental stressors. Future studies should incorporate concentration-response experiments and molecular analyses, such as genotoxicity and oxidative stress biomarkers, to further elucidate the mechanisms underlying the observed effects. Given that MC-LR and GLY frequently co-occur in freshwater systems impacted by agricultural runoff and harmful algal blooms, these results have important ecological relevance, as crayfish are likely chronically exposed and may accumulate contaminants in their tissues. Hepatopancreatic dysfunction can impair digestion, nutrient absorption, and energy storage, ultimately reducing growth and reproductive capacity and increasing vulnerability to other stressors [52]. Because crayfish are keystone species and ecosystem engineers, such impairments may also cascade through benthic food webs by altering nutrient cycling, organic matter processing, and the trophic transfer of energy and contaminants to higher trophic levels. Overall, these findings highlight the importance of assessing contaminant mixtures rather than individual stressors to better understand ecological risk in aquatic environments.

## Data Availability

Data, R Markdown (.Rmd) files, scripts, and associated analysis files used for data processing, statistical analyses, and figure generation reported in the manuscript and analysis outputs are available in a repository and can be accessed via https://doi.org/10.7910/DVN/OVXPWT.

## Supplementary Materials

The following supporting information can be downloaded at: https://www.mdpi.com/article/10.3390/toxics14080712/s1. Supplemental Materials include one table that provides data regarding the assessment of exposure concentrations referenced in the main text and flow cytometry scatter plots supporting the findings of this study. Table S1. LCMS data for random experimental samples and stock solutions. Figure S1. Forward scatter (FSC) and side scatter (SSC) FDA (A) and PI (B) fluorescence intensity plots from representative samples of control, MC-LR, GLY, and MC-LR + GLY treatment groups.

## Abbreviations

The following abbreviations are used in this manuscript:

CAT	Catalase
DMSO	Dimethyl sulfoxide
EPSPS	5-enoylpyruvylshikimate-3-phosphate synthase
FDA	Fluorescein diacetate
GLY	Glyphosate
GLY-d_2_	Deuterated glyphosate
GST	Glutathione-S-transferase
HABs	Harmful algal blooms
H&E	Hematoxylin and eosin
LC	Liquid chromatography
LC-MS	Liquid chromatography–mass spectrometry
MC-LR	Microcystin with Leucine and Arginine
MRM	Multiple reaction monitoring (MRM)
MS1 res	Mass analyzer 1 resolution
MS2 res	Mass analyzer 2 resolution
PBS	Phosphate-buffered saline
PI	Propidium iodide
