# Supplementary material for: Exposure to the Environmental Toxins Microcystin-LR and Roundup® Causes Cellular Damage in the Hepatopancreas of the White River Crayfish (Procambarus acutus)
Source: Toxics. 2026 Aug 12;14(8):712. doi: 10.3390/toxics14080712 (PMC13517703; doi:10.3390/toxics14080712)
Supplement: Supplementary file 1 [file toxics-14-00712-s001.zip › toxics-4414768-supplementary.pdf]

Supplementary Materials:

Table S1. LCMS data for random experimental samples and stock solutions.

Sample	[MC-LR] (ppb)	[Glyphosate] (ppb)
MC-LR Stock 1	3092	Not Detected
MC-LR Stock 2	3230	Not Detected
MC-LR Stock 3	3172	Not Detected
Mean (± S.D.)	3164.7 ± 69.3	Not Detected
GLY Stock 1	Not Detected	15151
GLY Stock 2	Not Detected	15227
GLY Stock 3	Not Detected	15443
**Mean (± S.D.)**	**Not Detected**	**15273 ± 151.5**
MC-LR Treatment 1	14.8	Not Detected
MC-LR Treatment 2	16.9	Not Detected
MC-LR Treatment 3	15.5	Not Detected
MC-LR Treatment 4	15.6	Not Detected
MC-LR Treatment 5	16.3	Not Detected
**Mean (± S.D.)**	**15.8 ± 0.8**	**Not Detected**
GLY Treatment 1	Not Detected	71.1
GLY Treatment 2	Not Detected	73.8
GLY Treatment 3	Not Detected	74.4
GLY Treatment 4	Not Detected	74.2
GLY Treatment 5	Not Detected	74.4
**Mean (± S.D.)**	**Not Detected**	**73.6 ± 1.4**
GLY and MC-LR Treat 1	15.9	71.6
GLY and MC-LR Treat 2	15.0	71.0
GLY and MC-LR Treat 3	15.8	75.3
GLY and MC-LR Treat 4	15.6	72.2
GLY and MC-LR Treat 5	16.6	70.6
**Mean (± S.D.)**	**15.8 ± 0.6**	**72.1 ± 1.9**
Control Stock 1	Not Detected	Not Detected
Control Stock 2	Not Detected	Not Detected
Control Stock 3	Not Detected	Not Detected
**Mean (± S.D.)**	**Not Detected**	**Not Detected**
Control Treatment 1	Not Detected	Not Detected
Control Treatment 2	Not Detected	Not Detected
Control Treatment 3	Not Detected	Not Detected
Control Treatment 4	Not Detected	Not Detected
Control Treatment 5	Not Detected	Not Detected
**Mean (± S.D.)**	**Not Detected**	**Not Detected**

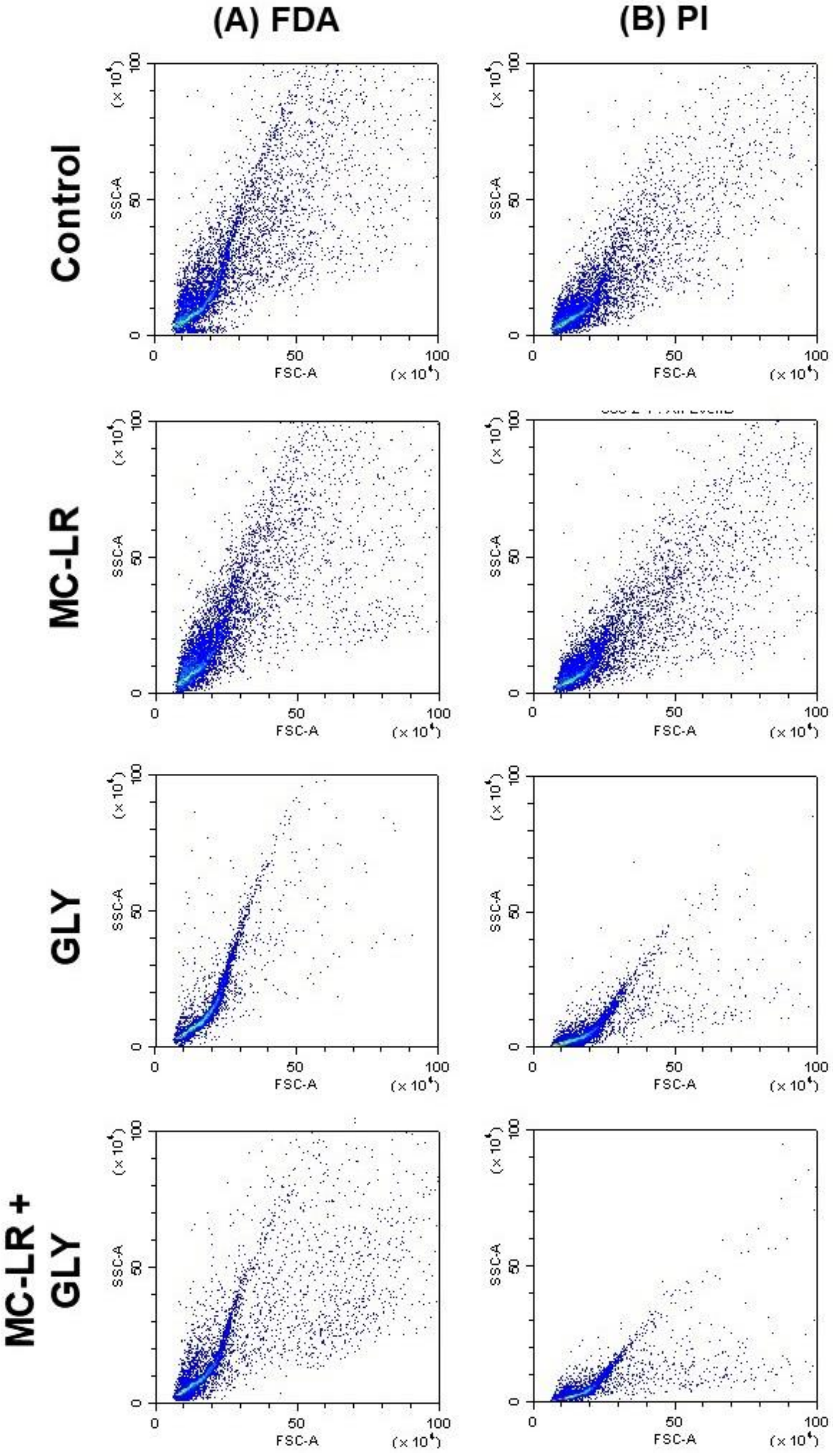

Figure S1. Forward scatter (FSC) and side scatter (SSC) FDA (A) and PI (B) fluorescence intensity plots from representative samples of control, MC-LR, GLY, and MC-LR + GLY treatment groups.
